# Inadequate care and excessive overprotection during childhood are associated with the presence of diabetes mellitus in adulthood in a general Japanese population: a cross-sectional analysis from the Hisayama Study

**DOI:** 10.1186/s12902-023-01474-4

**Published:** 2023-10-12

**Authors:** Mao Shibata, Masako Hosoi, Kozo Anno, Naoki Hirabayashi, Yoichiro Hirakawa, Hiroshi Kawata, Rie Iwaki, Ryoko Sawamoto, Nobuyuki Sudo, Toshiharu Ninomiya

**Affiliations:** 1 Department of Psychosomatic Medicine, Kyushu University Hospital, Fukuoka, Japan; 2 Department of Epidemiology and Public Health, Graduate School of Medical Sciences, Kyushu University, Fukuoka, 812-8582 Japan; 3 Division of Research Management, Center for Cohort Studies, Graduate School of Medical Sciences, Kyushu University, Fukuoka, Japan; 4 Department of Psychosomatic Medicine, Graduate School of Medical Sciences, Kyushu University, Fukuoka, Japan; 5 Department of Medicine and Clinical Science, Graduate School of Medical Sciences, Kyushu University, Fukuoka, Japan

**Keywords:** Diabetes, Parenting, Care, Overprotection, Attachment, Population study

## Abstract

**Objective:**

To investigate associations between parenting styles during childhood and diabetes in adulthood in a Japanese community.

**Methods:**

In 2011, 710 community-dwelling Japanese residents aged ≥ 40 years were assessed for the presence of diabetes and for their perceptions of the parenting style of their parents, as measured using the “care” and “overprotection” scales of the Parental Bonding Instrument. Care and overprotection scores for each parent were dichotomized by age-specific median values. Diabetes mellitus was defined as a fasting plasma glucose level of ≥ 7.0 mmol/L, a 2-h post-loaded glucose level of ≥ 11.1 mmol/L, HbA1c ≥ 6.5%, and/or the current use of insulin or oral glucose-lowering agents. The odds ratios (ORs) for prevalent diabetes were calculated using a logistic regression model.

**Results:**

The prevalence of diabetes was 14.9%. Subjects with a high paternal overprotection score had a significantly greater likelihood of prevalent diabetes than those with a low paternal overprotection score after adjusting for confounders (OR 1.71, 95% confidence interval [CI] 1.06–2.77), while there was no significant association between paternal care and diabetes. Additionally, the multivariable-adjusted ORs for the presence of diabetes were significantly higher in subjects with a low maternal care score (OR 1.61, 95%CI 1.00–2.60) or in subjects with a high maternal overprotection score (OR 1.73, 95%CI 1.08–2.80). Moreover, the subjects with a low care score and high overprotection score for both their father and mother had a significantly higher multivariable-adjusted OR of diabetes than those with a high care score and low overprotection score for both parents (OR 2,12, 95%CI 1.14–3.95).

**Conclusions:**

This study suggests that inadequate care and excessive overprotection during childhood may contribute to the development of diabetes in adulthood.

**Supplementary Information:**

The online version contains supplementary material available at 10.1186/s12902-023-01474-4.

## Background

Diabetes has emerged as a major health problem. In 2019 the World Health Organization listed diabetes as one of the top 10 leading causes of both death and disability-adjusted life years globally [1], and in 2017 about 500 million people were estimated to be suffering from diabetes worldwide [2]. Therefore, prevention of diabetes is regarded as a public health priority.

Childhood is widely acknowledged to play an important role in the formation of lifestyles and eating behaviors in adulthood [3]. Previous studies have reported that parenting styles affect obesity and food consumption in children [4, 5]. Accumulating evidence indicates that perceived parental attitudes and behaviors, especially “affectionless control,” a style characterized by the combination of insufficient care and excessive overprotection as measured by the Parental Bonding Instrument (PBI) [6], are associated with an increased risk of obesity and eating disorders [7, 8]. Affectionless control has been proposed to be a maladaptive form of parenting that results in a particular vulnerability to the occurrence of psychopathology [9, 10]. Additionally, childhood has been considered to be a critical period for the development of hormonal reactions related to stress resistance and blood glucose homeostasis [11]. Therefore, it could be assumed that parenting styles affect the future onset of diabetes. However, there are no community-based studies addressing the relation between parenting styles in childhood and the prevalence of diabetes in adulthood.

The aim of the present study was to examine the association between parenting styles, particularly perception of insufficient care and overprotection during childhood, and diabetes in adulthood in a general Japanese population.

## Methods

### Study participants

The Hisayama Study is an ongoing, long-term cohort study conducted to examine cardiovascular disease and its risk factors in Hisayama, a suburban town adjoining the metropolitan area of Fukuoka City in southwestern Japan. Health check-up surveys have been performed annually in Hisayama since 1961 [12, 13]. The present study was conducted as a cross-sectional sub-study of the Hisayama Study. Participants for the sub-study were recruited in 2011. Among the 2,250 residents aged 40 years or older who participated in the health check-up in 2011, a total of 793 subjects consented to participate in the present study. After excluding 78 residents without available data for both parents on the parenting questionnaire and 5 residents without a fasting blood test, the remaining 710 subjects (270 men and 440 women) were enrolled.

### Assessment of diabetes mellitus

Blood samples were collected from an antecubital vein after overnight fasting. Diabetes mellitus was determined by a 75 g oral glucose tolerance test in 450 subjects (63%) and by only a fasting blood sample in 260 subjects. Blood glucose was measured by the hexokinase method. Hemoglobin A1c (HbA1c) was measured by high performance liquid chromatography. A self-administered questionnaire including items on the use of oral glucose-lowering agents and insulin was completed by each participant and was checked by trained interviewers at the screening [12]. Diabetes mellitus was defined as a fasting plasma glucose level of ≥ 7.0 mmol/L (126 mg/dL), a 2-h post-loaded glucose level of ≥ 11.1 mmol/L (200 mg/dL), HbA1c (NGSP) ≥ 6.5% (48 mmol/mol IFCC), and/or the current use of insulin or oral glucose-lowering agents, according to the American Diabetes Association criteria in 2010 [14].

### Assessment of parental bonding

Perceived parenting styles were measured using the Parental Bonding Instrument (PBI), a self-report questionnaire with 25 items that measures parenting styles in the first 16 years of life, as recalled by the respondents [6]. The PBI is scored separately for the father and mother to evaluate the relationship between the respondent and each parent, as they are subjectively perceived. The respondents are asked to score their parents’ attitudes and behaviors separately, using a 4-point Likert scale. Two subscales of parenting style are measured by the PBI: care and overprotection. The “care” subscale reflects perceived parental emotional warmth, empathy, and closeness contrasted with coldness and rejection. The “overprotection” subscale reflects perceived parental over-control and intrusion contrasted with allowance of independence and autonomy. Each score of care and overprotection for each parent was dichotomized by the age-specific median values of each score, as shown in Supplemental Table S1. In addition, the parenting styles were also divided into four categories (PBI quadrants) by combining the dichotomized care and overprotection scores: “optimal bonding” (high care, low overprotection), “neglectful parenting” (low care, low overprotection), “affectionate constraint” (high care, high overprotection), and “affectionless control” (low care, high overprotection). The PBI score reflects the actual parenting attitude and is based on studies using corroborative witnesses and independent observers [15, 16]. The PBI has long-term stability [17] and its subscales have a high level of test–retest reliability and internal consistency [18]. The Japanese version of the PBI has been shown to have adequate validity [19].

### Measurement of potential confounding factors

A self-administered questionnaire concerning marital status, education, subjective economic status, habitual smoking, habitual drinking, habitual exercise, the use of medications (i.e., antihypertensive agents, oral glucose-lowering agents and insulin), and paternal and maternal history of diabetes was completed by each participant and was checked by trained interviewers at the screening [12]. Subjective economic status was assessed by a question asking, “How difficult or easy is your current financial status?” [20]. Response options for this question were “Very hard”, “Hard”, “Normal”, “Easy” and “Very easy”. Based on the participant's response, the subjective economic status was divided into low (very hard or hard) and high (normal, easy or very easy). Body height and weight were measured in light clothing without shoes, and the body mass index (kg/m^2^) was calculated. Blood pressure was measured three times after the subject had rested for at least five minutes in the sitting position. The mean of the three measurements was used for the present analysis. Hypertension was defined as a systolic blood pressure ≥ 140 mmHg, diastolic blood pressure ≥ 90 mmHg, and/or current use of antihypertensive agents [21]. Serum total cholesterol, high-density lipoprotein (HDL) cholesterol, triglyceride levels and cortisol were measured enzymatically.

### Statistical analysis

We divided the participants into high or low care and overprotection groups based on the age-specific median values of the care and overprotection scores in order to control for the confounding caused by the difference in the scores among generations. Comparisons of characteristics between the high and low parenting subscales (care and overprotection) were performed by an analysis of covariance (ANCOVA) for continuous variables or a logistic regression analysis for dichotomous variables with adjustment for age and sex. Odds ratios (ORs) and the confidence interval (CI) of the parenting styles on the presence of diabetes were estimated by logistic regression analysis. In the multivariable-adjusted analyses, the risk estimates were adjusted for age (continuous), sex (men or women), paternal and maternal history of diabetes (yes or no), marital status (with or without a partner), educational level (≤ 9 years or > 9 years), subjective economic level (low or high), lifestyle factors (i.e., hypertension [yes or no], serum total cholesterol [continuous], serum HDL cholesterol [continuous], serum triglycerides [log-transformed, continuous], BMI [continuous], current smoker [yes or no], current drinker [yes or no] and habitual exercise [yes or no]) and serum cortisol (continuous). To account for the interaction between the care and overprotection subscales, paternal and maternal bonding were classified into four quadrants: “high care, low overprotection” (optimal bonding) as a reference, “low care, low overprotection” (neglectful parenting), “high care, high overprotection” (affectionate constraint), and “low care, high overprotection” (affectionless control). Additionally, in order to assess the combined influence of the “affectionless control” parenting style by the father and mother on the presence of diabetes, parents’ parenting styles were classified into four quadrants as shown in Supplemental Table S2: “optimal bonding (high care and low overprotection) for both father and mother” as a reference, “affectionless control (low care and high overprotection) for either father or mother”, “affectionless control for both father and mother” and “other combinations of paternal and maternal parenting styles”. We also performed a sensitivity analysis on the association between the parenting styles and the presence of diabetes after including the 19 participants with a single mother and 3 participants with a single father. Finally, we performed an analysis to compare the characteristics between included and excluded subjects (*n* = 1539) by a Student’s* t*-test for parametric continuous variables, a Mann–Whitney U test for non-parametric ordinal variables, or a Chi-square test for dichotomous variables. All statistical analyses were performed using SAS software (version 9.4; SAS Institute, Cary, NC, USA). Statistical significance was defined as a two-tailed *P*-value of < 0.05.

## Results

Table 1 summarizes the age- and sex- adjusted characteristics of all participants according to the level of each parental care and overprotection score for the father and mother. Compared to women, men were more frequently distributed in the lower care group and higher overprotection group for both their father and mother. Subjects with a low level of care or high level of overprotection from their father and mother were significantly more likely to have low subjective economic level than those who received the opposite level of each parenting style. Additionally, subjects with a low level of care from their mother were significantly more likely to have a low educational level than those with a high level of care.

**Table 1:** Age- and sex-adjusted characteristics of participants according to the level of parental care and overprotection of the parent

	Father				Mother			
	Care		Overprotection		Care		Overprotection	
	High	Low	Low	High	High	Low	Low	High
	(*n* = 374)	(*n* = 336)	(*n* = 332)	(*n* = 378)	(*n* = 370)	(*n* = 340)	(*n* = 344)	(*n* = 366)
*Sociodemographic factors*								
Age, mean (SE)	60.1 (0.6)	59.3 (0.6)	60.2 (0.6)	59.4 (0.6)	59.9 (0.6)	59.6 (0.6)	59.7 (0.6)	59.7 (0.6)
Sex, male, %	28.2	48.9**	33.5	41.8*	31	45.6**	34.0	41.7*
Paternal history of diabetes, %	9.6	11.1	10.1	10.6	10.2	10.5	10.4	10.3
Maternal history of diabetes, %	7.6	7.8	7.5	7.8	7.2	8.3	7.8	7.6
Marital status, without partner, %	17.2	19.3	17.1	19.1	15.5	21.2	17.6	18.8
Educational level, under 10 years, %	8.8	11.4	9.1	10.9	7.9	12.4*	8.6	11.4
Subjective economic level, low, %	14.8	26.3**	14.4	25.3**	13.9	27.1**	15.9	24.3**
*Lifestyle and physical factors*								
Hypertension, %	41.0	40.3	40.4	41.0	39.8	41.7	36.8	44.4
Serum total cholesterol, mmol/l, mean (SE)	5.29 (0.05)	5.25 (0.05)	5.30 (0.05)	5.25 (0.05)	5.30 (0.05)	5.24 (0.05)	5.31 (0.05)	5.24 (0.05)
Serum HDL cholesterol, mmol/l, mean (SE)	1.72 (0.02)	1.69 (0.02)	1.72 (0.02)	1.70 (0.02)	1.69 (0.02)	1.73 (0.02)	1.71 (0.02)	1.70 (0.02)
Serum triglycerides, mmol/l, geometric mean (95%CI)	1.10 (1.05–1.16)	1.07 (1.01–1.13)	1.10 (1.05–1.17)	1.07 (1.02–1.13)	1.09 (1.04–1.15)	1.08 (1.02–1.14)	1.11 (1.05–1.17)	1.07 (1.02–1.13)
BMI, kg/m2, mean (SE)	22.8 (0.2)	22.9 (0.2)	23.0 (0.2)	22.7 (0.2)	22.9 (0.2)	22.8 (0.2)	22.8 (0.2)	22.8 (0.2)
Current smoker, %	6.0	7.9	6.7	7.1	6.7	7.1	6.4	7.4
Current drinker, %	54.8	55.1	56.2	53.8	57.6	52.0	55.7	54.2
Regular exercise, ≥ 3 times / week, %	12.0	14.2	13.9	12.3	14.0	12.0	15.0	11.2
Serum cortisol, nmol/L, mean (SE)	283 (5)	279 (5)	280 (5)	282 (5)	280 (5)	282 (5)	280 (5)	282 (5)

Values were tested by ANCOVA (for age, HDL cholesterol, BMI, cortisol and log-transformed triglycerides) or logistic regression model (for frequencies) with adjustment for age and sex. Age was sex-adjusted and sex was age-adjusted

Hypertension was defined as blood pressure ≥ 140/90 mmHg and/or use of an antihypertensive agent

^*^P < 0.05, **P < 0.01. Values are expressed as the age- and sex-adjusted mean (standard error [SE]) or frequency and geometric mean (95% CI)

**Fig. 1:**
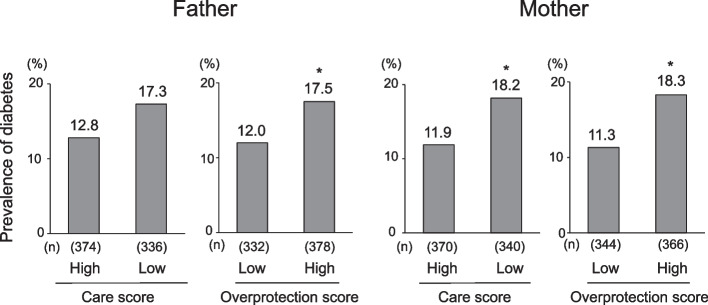
Crude prevalence of diabetes according to the level of parental care and overprotection. *P < 0.05 vs. optimal parenting

The median (IQR) fasting plasma glucose levels, prevalence of individuals with high blood glucose levels, and prevalence of the current use of insulin or oral glucose-lowering agents were 5.4 (4.6–6) mmol/L, 12.4% and 8.0%, respectively. The crude prevalence of diabetes was 15% (= 106/710) among all participants. The crude prevalence of diabetes was significantly higher in subjects with a high level of paternal overprotection, a low level of maternal care or a high level of maternal overprotection than in subjects with the opposite levels of each scale (Fig. 1). Table 2 shows the ORs for the presence of diabetes according to the level of care and overprotection for each parent. Subjects with a high level of paternal overprotection had a significantly greater likelihood of prevalent diabetes than those with a low level after adjustment for age and sex, sociodemographic factors, family history of diabetes, lifestyle factors, and serum cortisol (OR 1.71, 95% confidence interval [CI] 1.06–2.77), while there was no significant association between paternal care and diabetes. Additionally, the multivariable-adjusted ORs for the presence of diabetes were significantly higher in the subjects with a low level of maternal care (OR 1.61, 95%CI 1.00–2.60) or high level of maternal overprotection (OR 1.73, 95%CI 1.08–2.80) than in those with the opposite level of each scale. In a sensitivity analysis conducted after including the participants with a single parent, the findings were not changed substantially (Supplemental Table S3).

**Table 2:** Association of the score of parental care and overprotection with the presence of diabetes

	No. of diabetics	No. of participants	Age- and sex-adjusted		Multivariable-adjusted ^a)^	
			OR	(95%CI)	OR	(95%CI)
*Father*						
**Care**						
High	48	374	1.00	(Reference)	1.00	(Reference)
Low	58	336	1.28	(0.83–1.99)	1.27	(0.79–2.05)
**Overprotection**						
Low	40	332	1.00	(Reference)	1.00	(Reference)
High	66	378	1.55	(1.00–2.40)*	1.71	(1.06–2.77)*
*Mother*						
**Care**						
High	44	370	1.00	(Reference)	1.00	(Reference)
Low	62	340	1.57	(1.02–2.42)*	1.61	(1.00–2.60)*
**Overprotection**						
Low	39	344	1.00	(Reference)	1.00	(Reference)
High	67	366	1.69	(1.09–2.62)*	1.73	(1.08–2.80)*

*Abbreviations*: *OR* odds ratio, *CI* confidence interval

^*^ *P* < 0.05

^a)^ Adjusted for age, sex, paternal and maternal history of diabetes, marital status, educational level, subjective economic level, hypertension, serum total cholesterol, serum HDL cholesterol, serum triglycerides, BMI, current smoking, current drinking, habitual exercise and serum cortisol

**Fig. 2:**
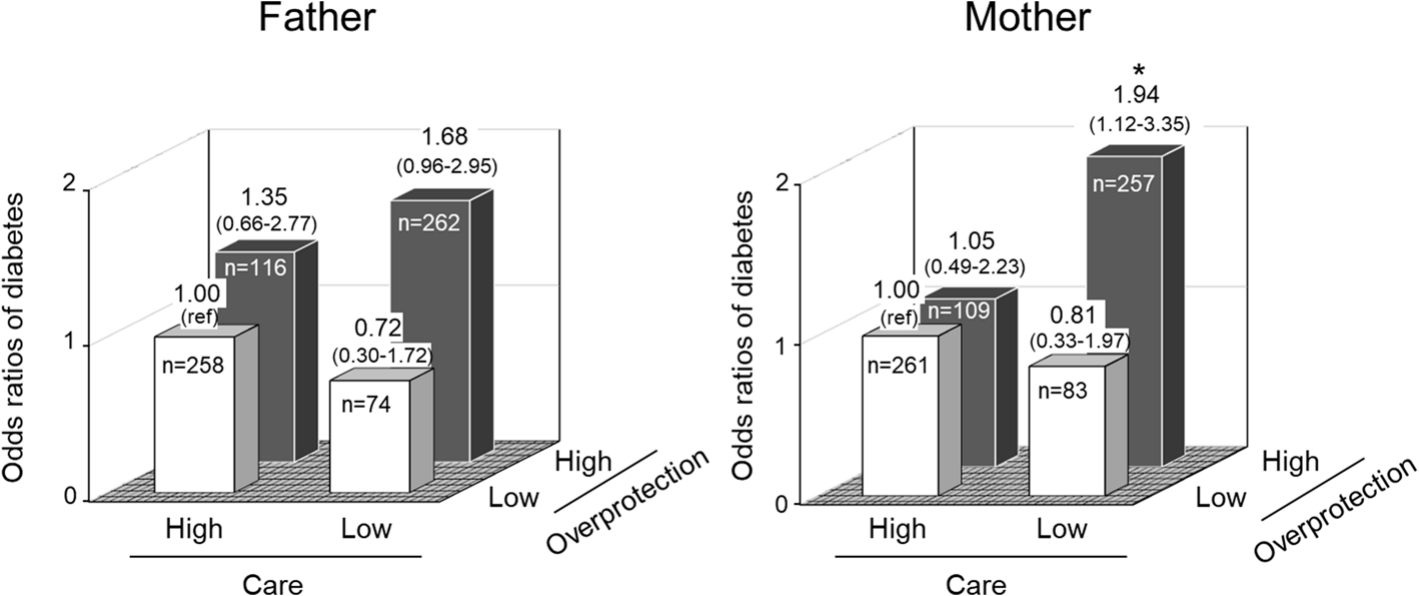
Multivariable-adjusted odds ratios of diabetes according to combined categories of parenting factors for each parent. Values are shown as odds ratios (95% confidence interval). *P < 0.05 vs. optimal parenting. The risk estimates were adjusted for age, sex, paternal and maternal history of diabetes, marital status, educational level, subjective economic level, hypertension, serum total cholesterol, serum HDL cholesterol, serum triglycerides, BMI, current smoker, current drinker, habitual exercise, and serum cortisol

Next, we examined the combined effects of low care and high overprotection on the presence of diabetes for each parent (Fig. 2). The multivariable-adjusted ORs for prevalent diabetes were significantly higher in subjects with both a maternal low care score and high overprotection score (affectionless control) than in those with both a high care score and low overprotection score (optimal bonding) (OR 1.94, 95%CI 1.12–3.35). A similar association was also observed for the paternal parenting style, but this association did not reach the level of statistical significance (OR 1.68, 95%CI 0.96–2.95).

Finally, we assessed the combined influence of an “affectionless control” parenting style by the father and mother on the presence of diabetes. The combinations of paternal and maternal parenting styles were classified into 4 categories (Supplemental Table S2). Consequently, the subjects with affectionless control by their father and mother had a significantly higher multivariable-adjusted OR of prevalent diabetes than those with the optimal bonding by both parents (OR 2.12, 95%CI 1.14–3.95). On the other hand, the ORs of diabetes for participants with affectionless control by either parent or with other combinations of bonding did not reach significance (Table 3).

**Table 3:** Combined influence of an “affectionless control” parenting style by the father and/or mother on diabetes

Combination of paternal and maternal parenting styles ^a^	No. of diabetics	No. of participants	Age- and sex-adjusted		Multivariable-adjusted ^b)^	
			OR	(95%CI)	OR	(95%CI)
High care and low overprotection for both the father and mother	24	205	1.00	(Reference)	1.00	(Reference)
Other combinations of paternal and maternal parenting styles	22	187	1.14	(0.65–2.13)	1.03	(0.52–2.04)
Low care and high overprotection for either the father or mother	17	117	1.35	(0.68–2.67)	1.23	(0.58–2.61)
Low care and high overprotection for both the father and mother	43	201	1.97	(1.12–3.47)*	2.12	(1.14–3.95)*

*Abbreviations*: *OR* odds ratio, *CI* confidence interval

^*^ *P* < 0.05

^a)^ Combinations of paternal and maternal parenting styles are shown in Supplemental Table 2

^b)^ Adjusted for age, sex, paternal and maternal history of diabetes, marital status, educational level, subjective economic level, hypertension, serum total cholesterol, serum HDL cholesterol, serum triglycerides, BMI, current smoking, current drinking, habitual exercise and serum cortisol

In the analysis comparing the characteristics between included and excluded subjects, we found that the excluded subjects were older, and showed higher frequencies of hypertension and current smoking, higher serum total cholesterol and BMI, and lower serum HDL cholesterol than the included subjects, whereas there was no clear difference in the prevalence of diabetes (Supplementary Table S4).

## Discussion

The present study demonstrated that paternal high overprotection, maternal low care and maternal high overprotection in childhood were associated with higher likelihood of the presence of diabetes in adulthood after adjusting for sociodemographic factors, family history, lifestyle factors and biological factors. Additionally, the parenting pattern of affectionless control by both parents significantly increased the risk of presence of diabetes compared to optimal bonding by both parents. These findings suggest that parenting styles during childhood, especially insufficient care and excessive overprotection (affectionless control), are related to diabetes in adulthood. On the other hand, the risk of the presence of diabetes was not increased in subjects with affectionless control by only one parent in the present study. This result suggests that a child’s future risk of diabetes could be decreased if at least one parent avoids the “affectionless control” parenting style.

As far as we know, there are no population-based epidemiological studies showing a significant association between parenting style and diabetes in adults. On the other hand, several hospital-based and population-based studies have shown a relationship between inadequate parenting, especially the “affectionless control” parenting style (i.e., low care and high overprotection), and the risk of eating disorders, obesity, and coronary heart disease [7, 8, 22, 23]. Since eating disorders and obesity are likely to be involved in the onset of diabetes, these previous findings may support ours. In the present study, moreover, low maternal care and parental overprotection were associated with diabetes, but there was no association between low paternal care and diabetes. Intriguingly, a previous study examining the association between parenting style and obesity found that only paternal care was not significantly associated with obesity [7]. In addition, previous studies examining mammalian childcare reported that mothers are significantly more likely to relate to their children than fathers during the breastfeeding stage [24]. The authors of that study stated that mothers have more frequent contact with their children at an early age compared to fathers. Therefore, this difference may impact the magnitude of the different results between fathers and mothers in regard to the association between care and diabetes. On the flip side, it is probable that high maternal care may have a protective effect against diabetes, as shown by the ORs of diabetes in participants parented with a high overprotection pattern (Fig. 2).

The exact mechanisms underlying the association between parenting styles and diabetes are not clear at present. However, we considered several possible mechanisms. First, there is the possibility that long-term stress from childhood affects diabetes. The “affectionless control” parenting style has been reported to be associated with the stress reaction, mental distress, lack of stress-coping skills, and interpersonal sensitivity [25–27]. The cumulative physiologic toll exacted on the body over time by efforts to adapt to life experiences, the so-called allostatic load, has been reported to result in prolonged responses to stress due to delayed shutdown of physiological reactivity [28, 29]. Long-term allostatic load from childhood to adulthood may lead to diabetes through activation of the hypothalamic–pituitary–adrenal (HPA) axis and/or sympathetic–adrenal–medullary (SAM) axis as stress responses [30, 31]. In the present study, however, the adjustment for serum cortisol, as an indicator of activity of the HPA-axis, did not attenuate the association between parenting style and diabetes. Therefore, another mechanism such as the activation of the SAM axis might underlie the association. Second, it is possible that eating behavior may have mediated the association between parenting style and diabetes. In the present study, the associations between parenting styles and diabetes were not altered substantially even after adjusting for lifestyle factors (e.g., smoking, drinking, habitual exercise and BMI) and family history of diabetes. However, some residual factors such as eating behavior and dietary pattern may mediate the association. Previous studies have reported that parental low care and parental low overprotection were associated with eating disorders such as bulimia nervosa and binge eating disorder [31, 32]. Maternal low care has been reported to be associated with emotional eating (i.e., eating driven by an emotional state) [31]. Chronic life stress has been reported to be associated with a greater preference for energy- and nutrient-dense foods, particularly those that are high in sugar and fat [33, 34]. An “affectionless control” parenting style might affect diabetes through those eating behaviors that cause increased blood glucose levels [35].

Parental low care refers to a parenting style where parents provide minimal emotional, physical, or financial support to their children. Parents who practice low care parenting may be emotionally distant or absent, neglectful, or indifferent to their children's well-being. Children who grow up with low care parents may struggle with issues such as low self-esteem, poor social skills, and difficulty forming healthy relationships [6, 36]. Overprotection refers to a parenting style in which a parent is excessively cautious and protective, to the point of limiting their child’s opportunities for growth, learning, and independence. Overprotection can manifest in different ways, such as preventing a child from taking risks or making decisions, shielding them from negative experiences, or not allowing them to engage in age-appropriate activities. Parental overprotection can have a range of effects on children, both in the short-term and long-term. These include lack of independence, anxiety and fear, low self-esteem, poor social skills and difficulty coping with adversity [6, 37]. There are many factors that can contribute to poor parental care and overprotection, including parental anxiety, parental history of abuse or neglect, cultural and societal expectations, unrealistic expectations of child development and lack of parenting skills and knowledge [38]. To avoid poor outcomes from parenting conducted under these limitations, it is important that parents be given support and appropriate resources to help them provide the best possible care and support for their children.

This study has several limitations. First, the ability to infer causality between the parenting style and diabetes was limited, because this study is a cross-sectional study. In this study, parenting styles in childhood were used as an exposure factor, but we could not deny the possibility that having diabetes might have an unfavorable impact on the scoring of past parenting styles. Prospective longitudinal studies are needed to clarify the contribution of inadequate parenting styles to the development of diabetes. Second, there is a possibility of selection bias, because only approximately one-third of individuals who attended the health check-ups consented to participate in the present study. In the analysis comparing the characteristics between included and excluded subjects, we found that the excluded subjects were older, and showed higher frequencies of potential confounding factors, whereas there was no clear difference in the prevalence of diabetes (Supplementary Table S4). Therefore, the results of the present study may have underestimated the association between parenting and diabetes. Third, the influence of residual confounders (e.g., lack of social support, serious life events, economic status in childhood, eating behavior, and dietary patterns) may still be present, although we did account for a wide range of confounders. In addition, the involvement of stress-related factors in adulthood in the association between parenting style and diabetes could not be examined because, with the exception of serum cortisol levels, the requisite data were not available. Lastly, we urge caution in generalizing our findings to populations with different backgrounds, since participants were recruited from a single town in Japan.

## Conclusions

The findings of the present suggest that inadequate care and excessive overprotection during childhood are related to diabetes in adulthood and that the association is much more significant when the inadequate parenting is received from both parents. Mass-education and social support for optimal parenting would thus be potential initiatives for the prevention of diabetes. Further prospective and interventional studies will be needed to clarify the mechanisms underlying the relation of parenting style to diabetes.

### Supplementary Information

**Additional file 1:** **Supplemental Table S1.** Parental care and overprotection scores for all participants according to age group. **Supplemental Table S2.** Combination of paternal and maternal parenting styles. **Supplemental Table S3.** Association of the score of parental care and overprotection with the presence of diabetes after including the participants with a single parent. **Supplemental Table S4.** Characteristics of included and excluded subjects.

## Data Availability

The datasets used in the current study are not publicly available because they contain confidential clinical data on the study participants. However, the data are available on reasonable request and with the permission of the Principal Investigator of the Hisayama Study, Toshiharu Ninomiya (Department of Epidemiology and Public Health, Graduate School of Medical Sciences, Kyushu University, Fukuoka, Japan).

## Abbreviations

OR: Odds ratio
CI: Confidence interval
PBI: Parental Bonding Instrument
HbA1c: Hemoglobin A1c
HDL cholesterol: High-density lipoprotein cholesterol
BMI: Body mass index
HPA-axis: Hypothalamic–pituitary–adrenal axis
SAM axis: Sympathetic–adrenal–medullary axis
