# Correction: Inadequate care and excessive overprotection during childhood are associated with the presence of Diabetes Mellitus in adulthood in a general Japanese population: a cross-sectional analysis from the Hisayama Study

**DOI:** 10.1186/s12902-023-01513-0

**Published:** 2023-11-21

**Authors:** Mao Shibata, Masako Hosoi, Kozo Anno, Naoki Hirabayashi, Yoichiro Hirakawa, Hiroshi Kawata, Rie Iwaki, Ryoko Sawamoto, Nobuyuki Sudo, Toshiharu Ninomiya

**Affiliations:** 1 Department of Psychosomatic Medicine, Kyushu University Hospital, Fukuoka, Japan; 2 Department of Epidemiology and Public Health, Graduate School of Medical Sciences, Kyushu University, Fukuoka, 812-8582 Japan; 3 Division of Research Management, Center for Cohort Studies, Graduate School of Medical Sciences, Kyushu University, Fukuoka, Japan; 4 Department of Psychosomatic Medicine, Graduate School of Medical Sciences, Kyushu University, Fukuoka, Japan; 5 Department of Medicine and Clinical Science, Graduate School of Medical Sciences, Kyushu University, Fukuoka, Japan

**Correction: BMC Endocr Disord 23, 222 (2023)**

**https://doi.org/10.1186/s12902-023-01474-4**

Following publication of the original article [1], the authors reported an error in the number of participants in Fig. 2.

The original Fig. 2 was:


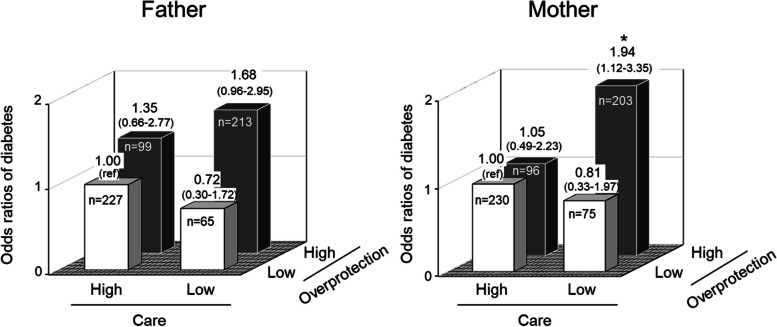


The updated Fig. 2 should read:


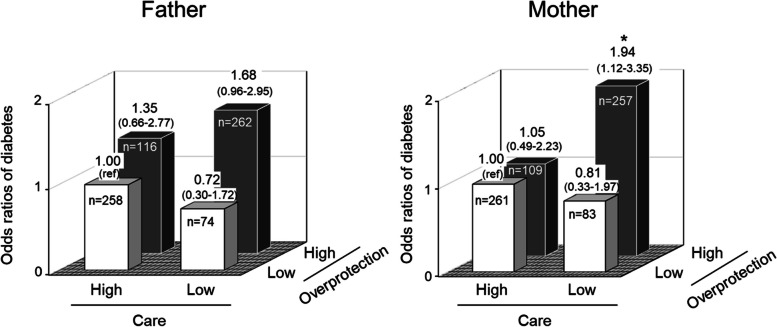


The original article [1] has been updated.
