# Supplementary material for: Inadequate care and excessive overprotection during childhood are associated with the presence of diabetes mellitus in adulthood in a general Japanese population: a cross-sectional analysis from the Hisayama Study
Source: BMC Endocr Disord. 2023 Oct 12;23:222. doi: 10.1186/s12902-023-01474-4 (PMC10568832; doi:10.1186/s12902-023-01474-4)
Supplement: Supplementary file 1 — Additional file 1: Supplemental Table S1. Parental care and overprotection scores for all participants according to age group. Supplemental Table S2. Combination of paternal and maternal parenting styles. Supplemental Table S3. Association of the score of parental care and overprotection with the presence of diabetes after including the participants with a single parent. Supplemental Table S4. Characteristics of included and excluded subjects. [file 12902_2023_1474_MOESM1_ESM.docx]

| Supplemental Table S1. Parental care and overprotection scores for all participants according to age group. | | | | | |
| --- | --- | --- | --- | --- | --- |
|  |  | Age group, years | | | |
|  |  | 40–49y | 50–59y | 60–69y | 70y and over |
|  |  | (n = 155) | (n = 182) | (n = 235) | (n = 138) |
|  | Score range | Median (IQR) | Median (IQR) | Median (IQR) | Median (IQR) |
| Father |  |  |  |  |  |
| Care | 0-36 | 27 (20-33) | 28 (22-33) | 28 (22-34) | 28 (21-34) |
| Over-protection | 0-39 | 9 (4-14) | 7 (4-13) | 8 (3-12) | 9 (4-14) |
| Mother |  |  |  |  |  |
| Care | 0-36 | 31 (25-34) | 30 (26-35) | 31 (26-36) | 31 (25-35) |
| Over-protection | 0-39 | 10 (5-14) | 8 (4-12) | 7 (3-12) | 8 (4-15) |

| Supplemental Table S2**.** Combination of paternal and maternal parenting styles | | | | | |
| --- | --- | --- | --- | --- | --- |
|  | | Mother | | | |
|  |  | Optimal bonding  *(high care + low overprotection)* | Neglectful parenting  *(low care + low overprotection)* | Affectionate constraint  *(high care + high overprotection)* | Affectionless control  *(low care + high overprotection)* |
| Father | Optimal bonding  *(high care + low overprotection)* | Optimal bonding by both the father and mother | Other combinations of paternal and maternal parenting styles | | Affectionless control by either the father or mother |
|  | Neglectful parenting  *(low care + low overprotection)* |  |  |  |  |
|  | Affectionate constraint  *(high care + high overprotection)* |  |  |  |  |
|  | Affectionless control  *(low care + high overprotection)* | Affectionless control by either the father or mother | | | Affectionless control by both the father and mother |

| Supplemental Table S3. Association of the score of parental care and overprotection with the presence of diabetes after including the participants with a single parent. | | | | | | | |
| --- | --- | --- | --- | --- | --- | --- | --- |
|  | No. of diabetics | No. of participants | Age- and sex-adjusted | |  | Multivariable-adjusted ^a)^ | |
|  |  |  | OR | (95%CI) |  | OR | (95%CI) |
| *Father* |  |  |  |  |  |  |  |
| Care |  |  |  |  |  |  |  |
| High | 48 | 374 | 1.00 | (Reference) |  | 1.00 | (Reference) |
| Low | 58 | 339 | 1.27 | (0.82-1.96) |  | 1.24 | (0.77-2.00) |
| Overprotection | |  |  |  |  |  |  |
| Low | 40 | 332 | 1.00 | (Reference) |  | 1.00 | (Reference) |
| High | 66 | 380 | 1.54 | (0.996-2.39) |  | 1.68 | (1.04-2.72)* |
| *Mother* |  |  |  |  |  |  |  |
| Care |  |  |  |  |  |  |  |
| High | 45 | 376 | 1.00 | (Reference) |  | 1.00 | (Reference) |
| Low | 66 | 353 | 1.57 | (1.03-2.41)* |  | 1.55 | (0.97-2.46) |
| Overprotection | |  |  |  |  |  |  |
| Low | 41 | 350 | 1.00 | (Reference) |  | 1.00 | (Reference) |
| High | 70 | 379 | 1.63 | (1.06-2.50)* |  | 1.65 | (1.04-2.62)* |
| Abbreviations: OR, odds ratio; CI, confidence interval.  * *p* < 0.05. a) Adjusted for age, sex, paternal and maternal history of diabetes, marital status, educational level, subjective economic level, hypertension, serum total cholesterol, serum HDL cholesterol, serum triglycerides, BMI, current smoking, current drinking, habitual exercise and serum cortisol. | | | | | | | |

| Supplemental Table S4. Characteristics of included and excluded subjects. | | | |
| --- | --- | --- | --- |
|  | Included subjects | Excluded subjects | *P* value |
|  | (n = 710) | (n = 1539) |  |
| Diabetes, % | 14.9 | 17.4 | 0.14 |
| *Sociodemographic factors* |  |  |  |
| Age, mean (SE) | 59.7 (0.4) | 66.2 (0.3) | <0.001 |
| Sex, male, % | 38.0 | 45.2 | 0.002 |
| Marital status, without partner, % | 18.5 | 21.8 | 0.07 |
| Paternal history of diabetes, % | 11.3 | 5.3 | <0.001 |
| Maternal history of diabetes, % | 7.9 | 7.2 | 0.53 |
| *Lifestyle and physical factors* |  |  |  |
| Hypertension, % | 41.8 | 57.8 | <0.001 |
| Serum total cholesterol, mmol/l, mean (SE) | 5.27 (0.03) | 5.23 (0.02) | 0.37 |
| Serum HDL cholesterol, mmol/l, mean (SE) | 1.71 (0.02) | 1.65 (0.01) | 0.008 |
| Serum triglycerides, mmol/l, medians (IQR) | 1.05 (0.78-1.47) | 1.12 (0.80-1.54) | 0.02 |
| BMI, kg/m2, mean (SE) | 22.8 (0.1) | 23.1 (0.1) | 0.046 |
| Current smoker, yes, % | 11.0 | 14.9 | 0.012 |
| Current drinker, yes, % | 53.9 | 47.4 | 0.004 |
| Habitual exercise, <3 times / week, % | 13.7 | 12.0 | 0.27 |
| Values are expressed as the mean (standard error [SE]), median (Interquartile range [IQR]), or frequency.  Hypertension was defined as blood pressure ≥140/90 mmHg and/or use of an antihypertensive agent. Diabetes was defined as a fasting plasma glucose level of ≥7.0 mmol/L (126 mg/dL), and/or a 2-h post-loaded or casual glucose level of ≥11.1 mmol/L (200 mg/dL), HbA1c (NGSP) ≥6.5% (48 mmol/mol IFCC) and/or current use of insulin or oral glucose-lowering agents. Values were tested by t-test (for age), Chi-square test (for frequencies) or Mann-Whitney U test (for serum triglycerides). | | | |
